# Artificial Intelligence-Based 18F-FDG PET/CT Radiomics for Mediastinal Lymph Node Staging in Non-Small Cell Lung Cancer: A Systematic Review

**DOI:** 10.3390/diagnostics16132014

**Published:** 2026-06-27

**Authors:** Alessia-Stephania Rosian, Agneta-Maria Pusztai, Amalia Constantinescu, Gabriel-Aurel Rus, Cristian Oancea, Diana Manolescu

**Affiliations:** 1 Doctoral School, “Victor Babes” University of Medicine and Pharmacy Timisoara, Eftimie Murgu Square 2, 300041 Timisoara, Romania; alessia.rosian@umft.ro; 2 Department of Nuclear Medicine, “Victor Babes” University of Medicine and Pharmacy Timisoara, Eftimie Murgu Square No. 2, 300041 Timisoara, Romania; 3 Department of Radiology and Medical Imaging, “Victor Babes” University of Medicine and Pharmacy Timisoara, Eftimie Murgu Square No. 2, 300041 Timisoara, Romania; dmanolescu@umft.ro; 4 Faculty of Medicine, “Victor Babes” University of Medicine and Pharmacy Timisoara, Eftimie Murgu Square No. 2, 300041 Timisoara, Romania; gabriel.rus@rezident.umft.ro; 5 Clinical Hospital of Infectious Diseases and Pneumophthisiology “Dr. Victor Babeș”, Gheorghe Adam No. 13, 300226 Timisoara, Romania; oancea@umft.ro; 6 Center for Research and Innovation in Precision Medicine of Respiratory Diseases (CRIPMRD), “Victor Babes” University of Medicine and Pharmacy Timisoara, 300041 Timisoara, Romania; 7 Department of Pulmonology, “Victor Babes” University of Medicine and Pharmacy Timisoara, 300041 Timisoara, Romania

**Keywords:** artificial intelligence, radiomics, 18F-FDG PET/CT, non-small cell lung cancer, mediastinal lymph node staging, lymph node metastasis, machine learning, deep learning

## Abstract

**Background/Objectives:** Accurate staging of mediastinal lymph nodes is essential for therapeutic decisions and prognostic assessment in non-small cell lung cancer (NSCLC). This systematic review evaluates diagnostic performance, validation strategies, and clinical significance of artificial intelligence (AI)-based 18F-fluorodeoxyglucose (18F-FDG) positron emission tomography (PET/CT) radiomics models for mediastinal nodal staging in NSCLC. **Methods:** Systematic literature searching was conducted in PubMed, ScienceDirect, and Scopus according to the PRISMA 2020 guidelines. Eligible studies used radiomic or AI-based approaches for mediastinal lymph node (LN) evaluation in NSCLC, with histopathology as a reference standard. Extracted data included study design, cohort characteristics, imaging method, validation strategy, and diagnostic performance metrics. Methodological quality was assessed by the QUADAS-2 tool. **Results:** Thirteen studies were included which are mainly retrospective in their designs with cohort sizes varying between 87 and 3265 patients. Models evaluated on external or prospective validation cohorts generally showed lower performance compared with training or internal datasets. However, clinically significant discriminative ability has been preserved across heterogeneous populations. In studies that directly compared methods, composite models integrating radiomic features with clinical factors and conventional PET metrics, sometimes including deep learning-derived features, consistently outperformed radiomics-only models. Additionally, selected approaches addressing FDG-related false-positive uptake improved distinction between benign and metastatic mediastinal lymph nodes; this is reflected by reduced false-positive classifications plus higher specificity compared with conventional PET/CT interpretation. **Conclusions:** AI-based 18F-FDG PET/CT radiomics show a promising discriminative capacity for mediastinal nodal staging in NSCLC, especially when it is integrated with clinical and conventional imaging variables. Although the model performance remains clinically significant within independent validation cohorts, attenuation compared with training datasets is commonly observed. Methodological heterogeneity, predominantly retrospective study designs, and the scarcity of prospective multicenter validation currently limit routine clinical implementation.

## 1. Introduction

Lung cancer remains the leading cause of cancer deaths around the world [1]. Non-small cell lung cancer (NSCLC) accounts for approximately 85% of all cases. Despite advances in surgical, systemic, and radiation therapies, survival outcomes are strongly influenced by the accuracy of disease staging at diagnosis [2]. In this context, accurately assessing lymph node involvement is crucial. Nodal involvement directly affects tumor stage, surgical eligibility, and the need for neoadjuvant or adjuvant therapy [3].

Conventional imaging methods like computed tomography (CT) and 18F-FDG positron emission tomography/computed tomography (PET/CT) are commonly used to evaluate nodal status [4] However, distinguishing benign from malignant lymph nodes remains challenging before surgery, owing to the limited sensitivity and specificity of traditional criteria [5]. Using radiomics and artificial intelligence (AI) techniques on PET/CT images offers a non-invasive framework for extracting quantitative imaging features [6]. This approach aims to enhance the detection and characterization of lymph node metastasis (LNM) beyond standard visual analysis [7].

Radiomics represents a high-throughput quantitative imaging approach that extracts a large number of reproducible features from standard medical images, capturing tumor phenotype, heterogeneity, and microenvironment characteristics beyond visual assessment, with the aim of supporting diagnosis, prognosis, and treatment personalization [8]. Recent thoracic imaging studies have further highlighted the broader applicability of radiomics and machine-learning approaches for mediastinal lymph node characterization. For example, chest CT-based radiomics combined with machine-learning algorithms have been explored for differentiating mediastinal lymphadenopathy associated with hematologic malignancies from metastatic solid tumors, further supporting the diagnostic relevance of quantitative imaging analysis in thoracic oncology [9].

Although 18F-FDG PET/CT is seen as the standard imaging method for staging lymph nodes in NSCLC, its diagnostic accuracy remains debated [10]. False-positive results can occur from inflammation or granulomatous disease, while false-negative results may happen in cases with small-volume metastases, which limit its reliability [11]. Although radiomics and AI-based methods try to overcome these issues, published studies exhibit considerable methodological variability, different performance metrics, and inconsistent validation strategies [7]. As a result, there is still discussion about the actual value of radiomics models compared to traditional imaging standards and whether they are ready for clinical use [12].

Several studies have demonstrated the potential of PET/CT-based radiomics models combined with machine learning (ML) or deep learning algorithms to enhance the prediction of lymph node involvement in NSCLC patients [13]. For example, PET/CT–based radiomics classifiers have been developed that integrate metabolic and textural features to differentiate malignant from benign nodal uptake patterns, outperforming conventional criteria and aiding personalized decision-making. Recent studies from multiple centers have confirmed that radiomics methods are broadly applicable [14]. In particular, models that combine PET and CT scan features using machine learning have consistently performed well across different groups. This approach appears to improve the accuracy of predicting whether NSCLC has spread to mediastinal and hilar lymph nodes [15].

One specific diagnostic challenge in NSCLC is the evaluation of mediastinal lymph nodes. The dense network of lymphatic tissue in the mediastinum often shows elevated uptake of 18F-FDG because of non-malignant causes, such as infectious processes, inflammatory diseases, granulomatous diseases, like sarcoidosis or tuberculosis, and reactive changes brought on by smoking or previous treatments [16,17]. Because of this, mediastinal lymph nodes frequently show increased metabolic activity even in the absence of metastatic involvement, which increases the likelihood of false-positive results on standard 18F-FDG PET/CT [18]. On the other hand, mediastinal nodes with small-volume or micrometastatic disease may remain below the metabolic or spatial resolution of conventional imaging, leading to false-negative evaluations [7,19]. The reliability of visual interpretation and straightforward threshold-based criteria is severely limited by these overlapping metabolic patterns, especially in the preoperative context [20]. In this context, advanced analytical approaches capable of capturing subtle textural heterogeneity and complex spatial relationships, such as radiomics and artificial intelligence, are increasingly viewed as essential tools for improving discrimination between inflammatory and malignant mediastinal lymph nodes [21].

Furthermore, integrating radiomics with clinical data and employing explainability tools such as SHAP not only improves predictive accuracy but also enhances physician interpretability and trust in real-world clinical practice [7]. While significant progress has been made, further research is needed to establish standardized radiomics workflows to confirm the reliability of predictive models in real-world settings and evaluate how these tools influence routine clinical practice [22]. Successfully incorporating these advanced models into routine staging for NSCLC could facilitate more precise, individualized, and less invasive assessments of lymph node involvement [23].

An emerging trend in this field is the shift from conventional hand-crafted radiomics toward deep learning radiomics (DLR), in which convolutional neural networks extract discriminative representations directly from raw image pixels without manual feature engineering [7,15,24]. DLR approaches often capture sub-visual patterns invisible to expert readers and undetectable by classical radiomics pipelines [25], and recent studies suggest they may further improve diagnostic accuracy when integrated with clinical and metabolic variables [26].

The main goal of this systematic review is to offer a clear and critical summary of the evidence on radiomics and artificial intelligence-based 18F-FDG PET/CT models for predicting mediastinal LNM in NSCLC without invasive procedures [20]. By carefully evaluating diagnostic performance, method quality, and clinical use, this review aims to highlight the current strengths and weaknesses of these methods. Overall, the evidence shows that AI-driven PET/CT radiomics has great potential for improving mediastinal nodal staging accuracy [27]. However, more standardization, external validation, and prospective studies are needed before routine clinical use can be recommended [7].

## 2. Materials and Methods

This systematic review was conducted in accordance with the 2020 Preferred Reporting Items for Systematic Reviews and Meta-Analyses (PRISMA) guidelines (Supplementary Materials) [28]. The review was not prospectively registered in a public database. The research question was formulated using the PICO framework—population, index test, comparator, and outcome—as outlined below:

• Population (P): Patients diagnosed with non-small cell lung cancer (NSCLC);
• Index Test (I): Artificial intelligence and radiomics-based models derived from 18F-fluorodeoxyglucose positron emission tomography/computed tomography (18F-FDG PET/CT);
• Comparator (C): Histopathological nodal assessment as the reference standard;
• Outcomes (O): Diagnostic performance for nodal staging and detection of lymph node metastasis.

A systematic literature search was performed using PubMed, ScienceDirect, and Scopus databases, covering studies published over a five-year period, from January 2021 to February 2026. These databases were selected to ensure broad coverage of peer-reviewed biomedical literature relevant to oncologic imaging and artificial intelligence, encompassing both clinical and technical research domains. Zotero reference management software (version 7.0.32) was used to collect, compile, and organize all records retrieved from the databases and to remove duplicate entries.

The search strategy was developed to ensure high sensitivity in identifying studies evaluating 18F-FDG PET/CT–based artificial intelligence or radiomics approaches for nodal or mediastinal staging in NSCLC. Database-specific search strategies were applied.

For PubMed, the search string was constructed using the following free-text keywords:

(“non-small cell lung cancer” OR NSCLC) AND (“positron emission tomography computed tomography” OR “PET/CT” OR “18F-FDG PET/CT”) AND (“radiomics” OR “artificial intelligence” OR “machine learning” OR “deep learning”) AND (“nodal staging” OR “mediastinal lymph node” OR “lymph node metastasis”).

For ScienceDirect, a keyword-based search strategy was applied using the following string, adapted to comply with database-specific Boolean limitations:

(“non small cell lung cancer” OR NSCLC) AND (“18F-FDG PET/CT” OR “FDG PET/CT”) AND (radiomics OR “deep learning”) AND (“nodal staging” OR “lymph node metastasis”).

For Scopus, the following search query was applied:

TITLE-ABS-KEY ((NSCLC OR “non-small cell lung” OR “lung cancer” OR “lung carcinoma”) AND (radiomics OR “radiomic feature” OR “texture analysis” OR “image feature extraction”) AND (“PET/CT” OR “PET-CT” OR “PET CT” OR “FDG-PET” OR “18F-FDG”) AND (“mediastinal lymph node” OR “mediastinal node” OR “lymph node staging” OR “nodal staging” OR “N staging” OR “N2” OR “mediastinal staging”)) AND (EXCLUDE (DOCTYPE, “re”) OR EXCLUDE (DOCTYPE, “ch”) OR EXCLUDE (DOCTYPE, “ed”) OR EXCLUDE (DOCTYPE, “bk”) OR EXCLUDE (DOCTYPE, “no”) OR EXCLUDE (DOCTYPE, “sh”) OR EXCLUDE (DOCTYPE, “tb”) OR EXCLUDE (DOCTYPE, “le”)) AND (LIMIT-TO (SUBJAREA, “MEDI”)) AND (LIMIT-TO (LANGUAGE, “English”))

Eligible studies were selected based on the following inclusion criteria:

1. Studies including adult patients diagnosed with NSCLC;
2. Studies employing 18F-FDG PET/CT-based artificial intelligence or radiomics models;
3. Studies evaluating nodal or mediastinal staging or lymph node metastasis;
4. Studies reporting diagnostic or staging-related outcomes;
5. Full-text original research articles involving human participants, published within the predefined search period.

Exclusion criteria included:

1. Studies not focused on NSCLC or not evaluating 18F-FDG PET/CT–based artificial intelligence or radiomics approaches;
2. Studies using non-FDG PET tracers or CT-only or MRI-only imaging;
3. Studies focusing exclusively on prognostic or survival outcomes without diagnostic or staging evaluation;
4. Publications available only as abstracts, without accessible full text;
5. Non-original publications, including reviews, editorials, letters to the editor, case reports, conference proceedings, systematic reviews, or meta-analyses;
6. Animal or preclinical studies.

Titles and abstracts were screened independently by two reviewers. Full-text versions of potentially eligible studies were subsequently assessed according to the predefined inclusion and exclusion criteria. Any discrepancies during the screening and selection process were resolved through consensus. Data were extracted independently by two reviewers using a standardized data extraction table. Extracted information included author and year of publication, study design, patient population, imaging modality, artificial intelligence or radiomics methodology, target outcome related to nodal staging or lymph node metastasis, and reported diagnostic performance metrics, when available.

### 2.1. Quality Assessment of Diagnostic Accuracy Studies-2 (QUADAS-2)

The methodological quality and risk of bias of the included studies were assessed using the QUADAS-2 tool. Four domains were evaluated: patient selection, index test, reference standard, and flow, and timing. Each domain was judged as having low, unclear, or high risk of bias. The assessment was performed independently by two reviewers, with disagreements resolved by consensus. The results of the QUADAS-2 assessment are summarized in Table 1.

Ethical approval and informed consent were not required for this study, as it was based exclusively on previously published data. No original datasets, biological samples, or patient-level data were generated or analyzed. All materials and protocols associated with this review are available within the manuscript. Generative artificial intelligence tools (QuillBot, Learneo, Inc., Chicago, IL, USA) were not used for data generation, analysis, or interpretation. Their use was limited exclusively to language refinement and formatting assistance. The authors take full responsibility for the accuracy and integrity of all scientific content presented in this manuscript.

### 2.2. Study Selection

**Figure 1:**
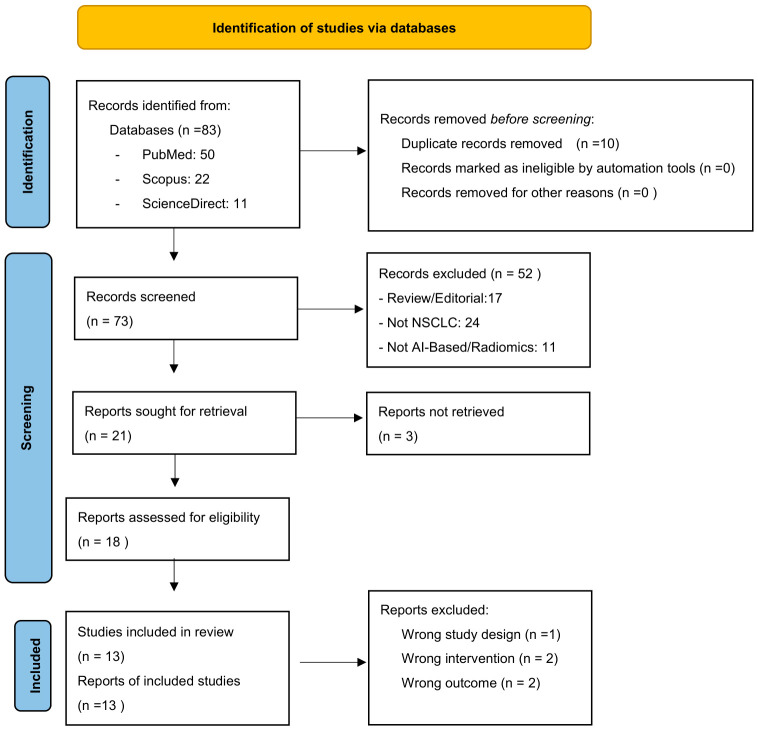
PRISMA 2020 flow diagram of the study selection process, including database contributions and reasons for exclusion.

A total of 83 records were identified through database searching (PubMed: *n* = 50; ScienceDirect: *n* = 11; Scopus: *n* = 22). After removal of duplicate records (*n* = 10), 73 studies were screened by title and abstract. Fifty-two records were excluded during screening. Twenty-one reports were sought for full-text retrieval, of which three were not retrievable. Eighteen full-text articles were assessed for eligibility. Five studies were excluded due to inappropriate study design (*n* = 1), intervention (*n* = 2), or outcome (*n* = 2). Ultimately, Figure 1 describes the PRISMA 2020 flow diagram for the thirteen studies included in the systematic review.

## 3. Results

### 3.1. Characteristics of Included Studies

The thirteen included studies evaluated artificial intelligence–based PET/CT approaches for lymph node assessment in non-small cell lung cancer, using histopathology as the reference standard. All studies described in Table 2 were observational in design, predominantly retrospective, with total sample sizes ranging from 88 to 3265 patients when training and validation cohorts were combined. Cohort definitions varied across studies and included pre-treatment imaging populations, external validation datasets, and cohorts enriched for surgically treated patients in independent validation setting (Table 2).

### 3.2. Model Development and Predictive Performance

**Figure 2:**
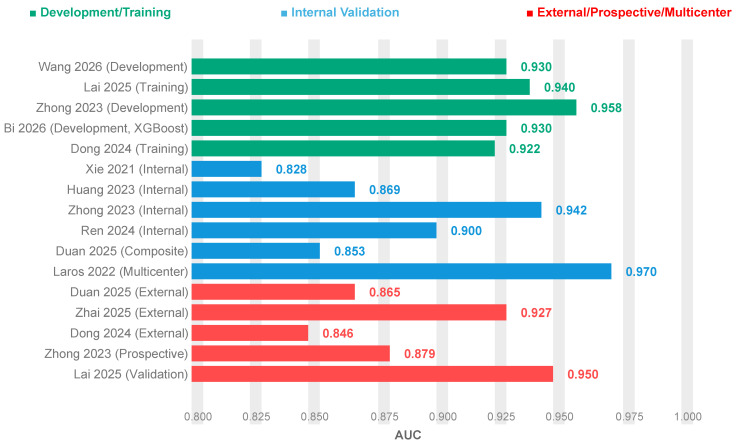
Reported AUC Values across included studies–Forest Plot [7,9,14,15,26,29,31,32,33,34,35,36].

Lai et al. (2025) [14] performed a retrospective study in which they developed and internally validated a PET/CT-derived radiomics model, which incorporates clinical, conventional metrics of imaging, and radiomic features to predict LNM. The authors reviewed multiple machine-learning models and found a random-forest-augmented logistic-regression model as the one with the best performance. Combined model area-under-the-curve (AUC) values in the training and independent validation groups, respectively, were 0.94 and 0.95; for occult LN dissemination, the AUCs were 0.89 (training) and 0.78 (validation) [14]. In another retrospective study, a PET/CT radiomics nomogram was developed on nodal staging (Xie et al., 2021) [29]. Variable radiomics data derived through CT aspect of PET/CT was added to maximum standardized uptake value (SUVmax) in a multivariable logistic regression model. The integrated nomogram had an AUC of 0.881 in the training set and 0.872 in an independent test set, which was better than the performance of the radiomics-only model. In their study, Wang et al. (2026) [9] compared PET/CT radiomics and machine-learning classifiers to predict lymph-node metastasis and N2 disease. As Figure 2 shows, the models developed had AUC of 0.93 with overall lymph-node metastasis, and 0.94 with N2-stage metastasis [22]. Furthermore, Bi et al. (2026) [36] retrospectively included 390 patients with NSCLC (including 1026 lymph node stations) and systematically compared the predictive performance of eight machine learning algorithms (logistic regression, CART, SVM, GBDT, random forest, MLP, XGBoost, and KNN) for mediastinal lymph node metastasis based on PET-CT and clinical pathology data. XGBoost consistently delivered the best discriminative performance with an AUC of 0.93, suggesting that the choice of model architecture plays a major role in prediction accuracy regardless of feature engineering approach [36].

### 3.3. Independent Validation and Cohort-Specific Performance

Wdowiak et al. (2025) [30] conducted independent validation on a previously created machine learning classifier which uses routine PET/CT measurements and clinical characteristics for distinguishing N0/1 from N2/3 disease. Two independent cohorts were analyzed. In the Charité cohort, specificity of the machine learning classifier was comparable to standard PET/CT criteria whereas in the TCIA cohort the classifier showed substantially higher specificity compared with the standard PET/CT assessment. The authors noted that one validation cohort included only surgical patients, a case-mix composition that may have influenced performance estimates [30]. Laros et al. (2022) [35] performed a multicenter retrospective analysis at 2 Western European centers, involving 148 patients with NSCLC, using an XGBoost classifier incorporating radiomic features from PET/CT and clinical data. Three modeling scenarios were evaluated: the node-only model achieved an AUC of 0.94, which improved to 0.97 upon inclusion of primary tumor radiomic features; adding clinical information on distant metastases did not confer further benefit, with all clinical feature combinations maintaining an AUC of 0.97; and feature selection analyses demonstrated that a reduced set of top features preserved equivalent discriminative performance (AUC 0.96–0.97), compared with 0.87 for conventional SUV thresholding. Performance in the external test cohort (center 2) was statistically significantly lower than in the internal validation set (center 1), confirming that model efficacy is influenced by center-specific patient selection and imaging protocols [35].

### 3.4. Performance of Deep Learning Models

**Figure 3:**
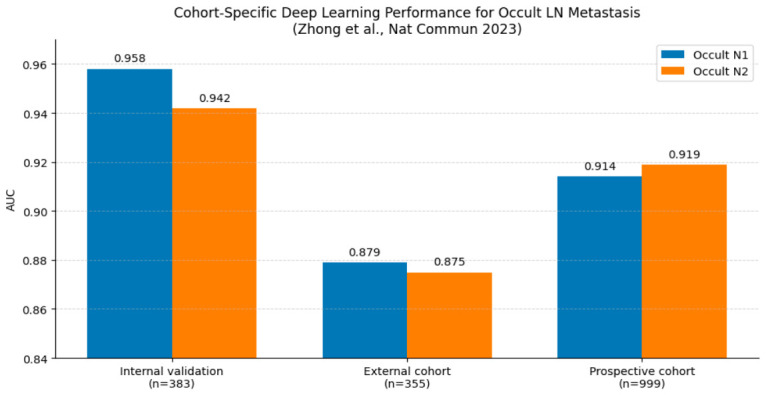
Cohort-specific AUC values of the deep learning nodal metastasis signature (Zhong et al., 2023) [26].

Zhong et al. (2023) developed a cross-modal deep learning nodal metastasis signature using PET/CT images to predict occult lymph node metastasis in non-small cell lung cancer with clinically normal nodes [26], as represented in Figure 3. It was trained on an internal cohort of 1911 patients and subsequently validated on an external cohort of 355 patients and a prospective cohort of 999 patients. In occult N1 metastasis, the area under the receiver operating characteristic curve (AUC) ranged between 0.879 and 0.958 across cohorts, but in occult N2 metastasis, the AUC values varied between 0.875 and 0.942.

Duan et al. (2025) compared retrospectively deep and machine-learning methods of predicting lymph node metastasis by training an XGBoost classification of a ResNet50 network output on a combination of clinical features, radiomic descriptors, and deep learning output. The resultant composite model was 0.853 AUC [15].

### 3.5. Multimodal Feature Integration

**Figure 4:**
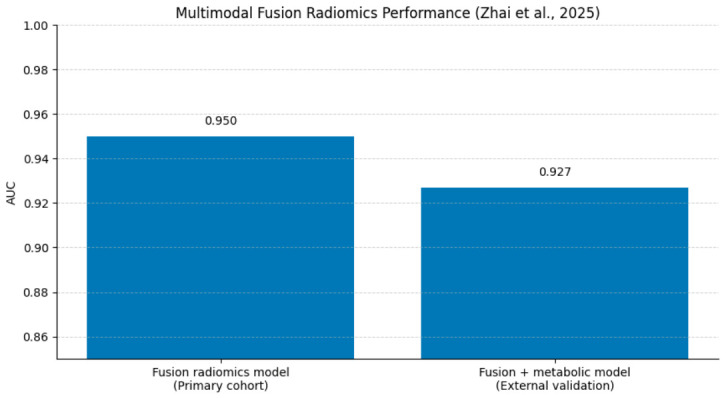
Multimodal fusion radiomics performance (Zhai et al., 2025 [32]).

Zhai et al. (2025) [32] conducted a multicenter retrospective study to establish and externally validate the multimodality fusion radiomics models based on PET/CT images to predict mediastinal-hilar lymph node metastasis. The fusion radiomics model and the augmented fusion radiomics plus metabolic parameter model had area under the curve (AUC) values of 0.950 and 0.927, respectively, in the primary cohort and external validation cohort respectively [32] (Figure 4).

### 3.6. Integrated Radiomics and Nomogram-Based Models

Huang et al. (2023) [33] developed a PET/CT radiomics signature integrated with a clinical nomogram to predict the mediastinal lymph node metastasis. The radiomics-only model achieved an AUC of 0.851 and 0.826 at two analytic partitions, compared with the integrated radiomics nomogram model that had AUC 0.869 and 0.847 [33].

### 3.7. False-Positive Lymph Node Classification

Ren et al. (2024) [31] conducted a focused analysis of the discrimination between the false-positive lymph nodes and true metastatic lymph nodes using PET/CT radiomic results. The radiomics models obtained false positive lymph node discrimination and prediction of lymph node metastasis area under the curve (AUCs) of 0.90 and 0.89 respectively [31]. Dong et al. (2024) [34] performed a multicenter retrospective analysis across three institutions, enrolling 381 patients with diagnostically confusing mediastinal lymph nodes on 18F-FDG PET/CT. The authors built a gradient-boosted decision tree–logistic regression (GBDT-LR) hybrid model with explicit feature interactions from PET/CT radiomic and clinical data. The GBDT-LR model attained an AUC of 0.90 in the training cohort and 0.846 in the external validation cohort, and it outperformed both stand-alone deep learning and conventional radiomics methods in identifying benign from malignant confused mediastinal lymph nodes [34].

### 3.8. Summary of Diagnostic Performance

All included studies demonstrated moderate to high diagnostic performance, with AUC values ranging from 0.828 to 0.958. Studies employing deep learning architectures or multimodal feature integration generally reported superior and more consistent results, particularly when evaluated on external or prospective validation cohorts. Multicenter investigations and systematic algorithmic comparisons further confirmed that model architecture, feature interaction strategies, and cohort diversity influence diagnostic performance independently of radiomic feature selection.

## 4. Discussion

### 4.1. Interpretation of Findings

This systematic review is a critical evaluation of the existing evidence on artificial intelligence-based 18F-FDG PET/CT radiomics for mediastinal LN staging in NSCLC, and in particular, methodological design, diagnostic accuracy, and reproducibility. The findings, when interpreted in the context of the included studies, support the working hypothesis that AI-based quantitative analysis may complement mediastinal nodal evaluation using standard PET/CT criteria provided that models are constructed using clinically relevant endpoints and are appropriately validated.

A number of studies that exclusively investigated mediastinal LN metastasis case showed that PET/CT radiomics-based models can attain high and reproducible diagnostic performance. One of the best validation results was recorded by Lai et al. (2025) with an AUC of 0.95 of mediastinal LN metastasis through a model combining radiomic features with clinical variables [14]. The consistency of the performance on an independent validation cohort indicates that the radiomics-based textural data represents patterns of biologic relevance and is not well represented by traditional PET measures. On the same note, Huang et al. (2023) demonstrated that PET/CT radiomics with a clinical nomogram was more discriminatory than radiomics-only models, indicating that clinical context provides complementary information on mediastinal nodal evaluation [33].

The methodological framework of earlier nomogram-based approaches provides important context for subsequent radiomics models. Xie et al. (2021) demonstrated improved preoperative nodal staging when CT-based radiomic features were combined with SUVmax, compared with conventional PET/CT interpretation alone [29]. However, the absence of a mediastinal-specific nodal endpoint in that analysis may partially explain the more moderate AUC values reported. Subsequent radiomics models explicitly targeting mediastinal lymph nodes can be viewed as an extension of these earlier concepts, applied to a diagnostically challenging nodal compartment [37].

The influence of endpoint definition is further illustrated by studies with mixed nodal outcomes. Wang et al. (2026) have found that AUC values for overall LN metastasis and N2 disease prediction were high, indicating that radiomics-based models can be especially informative in case of advanced nodal disease [9]. However, these findings are constrained by the fact that only heterogeneous nodal regions were considered, limiting generalizability to mediastinal-specific staging. In their work, Wdowiak et al. (2025) emphasized the vulnerability of model performance to cohort composition by confirming a machine-learning classifier on two independent datasets of varying case mixes [30]. Their results emphasize the point that the high diagnostic accuracy is not necessarily transportable and that the observed robustness may be due to selection bias or distribution of the reference standards and not inherent model generalizability.

Studies that used deep learning architectures in conjunction with substantial internal training and independent external or prospective validation demonstrated the most reliable indicators of reproducibility. Zhong et al. (2023) developed a cross-modal deep learning model, trained on a large internal cohort and validated on external and prospective datasets [26]. Despite maintaining high AUC values across cohorts, prospective validation revealed some performance attenuation, which reflects difficulties in practical clinical implementation. These results imply that deep learning models tend to maintain overall discriminating performance across a variety of populations, even though they may show decreased peak discrimination in heterogeneous contexts [38]. However, they still remain susceptible to clinical and technological heterogeneity. Similarly, Zhai et al. (2025) used a multicenter, multimodality fusion radiomics technique and found no significant changes in AUC between training and external validation cohorts, highlighting the significance of feature integration and multicenter validation in improving model generalizability [32].

Hybrid modeling approaches that combine deep learning-derived representations, clinical variables, and radiomic features offer an additional example of this trend. For the prediction of lymph node metastases, Duan et al. (2025) showed that integrating ResNet50-based deep features with radiomics and clinical data in an interpretable machine-learning framework produced diagnostic performance on par with less interpretable models [7]. Duan et al. (2025) demonstrated in a later externally validated study that the use of SHAP-based explainability maintained clinical interpretability without sacrificing diagnostic accuracy [15]. Together, these results imply that model transparency and multimodal integration may have a greater impact on robustness and generalizability in diverse clinical contexts than the slight increases in peak AUC seen in development cohorts. The significance of systematic algorithm comparison is underscored by an evaluation of eight machine-learning models on a uniform PET-CT dataset, which revealed that XGBoost consistently surpassed other classifiers. This indicates that the choice of model architecture significantly impacts predictive performance, independent of feature engineering [36].

Another dimension of clinical relevance is considered by the studies focusing on false-positive lymph nodes, which is the critical restriction of the traditional use of the 18F-FDG PET/CT at the mediastinum. As was demonstrated by Ren et al. (2023), the clinico-biological-radiomics models can be used to effectively differentiate between inflammatory and metastatic lymph nodes with a high diagnostic accuracy [31]. This finding directly addresses one of the most challenging situations when using mediastinal staging and supports the role of AI-based methods as complementary strategies when conventional visual interpretation is unreliable.

A significant limitation of AI-driven PET/CT radiomics models remains the challenge of achieving consistent diagnostic performance across heterogeneous clinical environments. In this context, external validation constitutes a critical prerequisite for assessing true model generalizability beyond development cohorts. Wdowiak et al. (2025) provided an important contribution by independently validating a machine-learning classifier based on routinely obtainable PET/CT parameters and clinical variables for mediastinal nodal staging in NSCLC [30]. Although inter-cohort variability in diagnostic performance was observed, clinically relevant discriminatory performance was maintained, underscoring the translational potential of AI-assisted nodal assessment. At the same time, these findings highlight the substantial influence of cohort composition, referral pathways, prevalence of nodal disease, and surgical selection bias on the transportability of predictive models [30]. Collectively, these observations reinforce the necessity for prospective multicenter validation frameworks before widespread clinical implementation can be justified. The results align with Laros et al. (2022), who illustrated in a Western European multicenter cohort that XGBoost-based classification of intrathoracic lymph nodes improved with the inclusion of primary tumor data, while also affirming that model efficacy is influenced by center-specific patient selection and imaging protocols [35].

Another major diagnostic challenge is represented by false-positive FDG uptake within mediastinal lymph nodes, particularly in the setting of inflammatory, infectious, or granulomatous diseases [16,17,18]. Conventional PET/CT interpretation remains susceptible to overestimation of nodal metastatic involvement because metabolically active benign lymphadenopathy frequently overlaps with malignant uptake patterns [16]. In this context, radiomics and machine-learning approaches appear particularly valuable because they may capture complex intranodal heterogeneity beyond conventional visual or threshold-based assessment. Ren et al. (2023) demonstrated that clinico-biological-radiomics models may substantially improve discrimination between inflammatory and metastatic lymph nodes, potentially reducing unnecessary invasive staging procedures and inappropriate therapeutic stratification [31]. Overall, these findings support the emerging role of AI-assisted PET/CT analysis as an adjunctive decision-support strategy in diagnostically indeterminate mediastinal staging scenarios where conventional imaging criteria remain insufficient. Dong et al. (2024) illustrated across three centers that a gradient-boosted decision tree model, which included feature interactions, surpassed both independent deep learning and traditional radiomics methods in distinguishing ambiguous mediastinal lymph nodes, thereby reinforcing the efficacy of hybrid machine-learning strategies in diagnostically challenging situations [34]. Furthermore, the biological underpinnings of occult nodal metastasis deserve closer integration with radiomics research. Occult metastases arise in lymph nodes that appear morphologically and metabolically normal on conventional imaging, yet frequently harbor molecular alterations such as EGFR mutations or elevated PD-L1 expression that reshape the nodal microenvironment [35,39]. Whether radiomics- or DLR-derived imaging phenotypes can capture these molecular features non-invasively remains an open question. Bridging this gap in future studies would allow a biology-informed approach to nodal staging beyond a merely morphometric or metabolic assessment.

In all the literature reviewed, among studies with external or prospective validation, it is a consistent finding that performance stability among validation cohorts is a more significant measure of clinical usefulness than maximal training-set accuracy. External or prospective validation studies that included external or prospective validation always demonstrated certain levels of performance attenuation but still provided clinically relevant discrimination. This trend can be traced in the studies of Zhong et al., Zhai et al., and Duan et al., indicating that AI-driven PET/CT radiomics is able to capture reproducible imaging phenotypes, though within the limitations of cohort heterogeneity and methodological variability [7,26,32,40]. Certain technical features within the radiomics workflow across the included investigations merit specific examination. Heterogeneity in image capture (e.g., changes in scanner manufacturers, reconstruction techniques, voxel dimensions and FDG injection-to-scan intervals) is an inherent source of non-biological variability that can systematically impact the derived radiomic characteristics. Segmentation methods varied from human delineation to semi-automated and completely automated deep learning based approaches, each adding certain operator or algorithm dependent biases that affect the stability of the feature. The selection of feature selection methods also differed without explicit justification: LASSO regularization [33], random-forest importance ranking [14], XGBoost-embedded selection [15] and end-to-end deep learning representations [7,26]. Crucially, none of the included studies performed formal assessment of reproducibility of radiomic features with test-retest or inter-observer variability analyses, limiting confidence in the robustness of the presented signatures and underscoring the need for standardized pipelines incorporating mandatory reproducibility assessment.

### 4.2. Limitations of the Present Review

Several limitations of this review warrant discussion in this review. First, the included studies are heterogeneous in nature and mainly retrospective and single-centered. Differences in patient cohorts, image acquisition parameters, segmentation methods, feature-extraction pipelines, and validation strategies preclude direct comparability across studies. The variations between the definition of nodal endpoints, particularly regarding mediastinal-specific staging, also affect reported diagnostic performance. The absence of standardized reporting structures limits the assessment of reproducibility and rigorous evidence synthesis.

This heterogeneity has a direct methodological consequence for the strength of the synthesized evidence: the inability to conduct a quantitative meta-analysis. A formal meta-analysis would have enabled statistical pooling of diagnostic accuracy over thousands of patients, providing a definitive pooled estimate for sensitivity, specificity, and AUC.

The predominantly retrospective design further limits causal inference and real-world translatability. Retrospective analyses are susceptible to selection bias, incomplete data, and case-mix distortions that may inflate diagnostic performance compared to prospective real-time validation. Among the 13 included investigations, only Zhong et al. (2023) [26] included a prospective validation cohort, which highlights the scarcity of real-time clinical evidence.

An additional concern is the persistent performance attenuation observed when AI models are evaluated on populations different from those used for development. This phenomenon, reported by Zhong et al. (2023) [26], Zhai et al. (2025) [32], and Wdowiak et al. (2025) [30], indicates vulnerability to variations in scanner manufacturers, acquisition protocols, patient demographics, and disease prevalence, raising serious concerns about generalizability and the readiness of current models for unsupervised deployment.

A further limitation concerns the size and geographic homogeneity of the included cohorts. Several studies relied on small datasets, Zhai et al. (2025) [32] with 88 patients and Wdowiak et al. (2025) [30] with 87 patients, limiting statistical power and increasing the risk of overfitting. AUC estimates from such samples have wide confidence intervals and are unlikely to replicate in larger populations. In contrast, even studies with large cohorts (>3000 patients, Zhong et al., 2023 [26]) were largely from single countries, mostly China. This regional concentration limits applicability to other healthcare systems with different imaging equipment, FDG dose protocols, and prevalence patterns of granulomatous or inflammatory disease (tuberculosis, sarcoidosis). The lack of cross-country and cross-continent validation therefore remains a major gap in the current evidence base.

### 4.3. Future Directions

In order to ensure actual model transportability, future clinical studies should concentrate on prospective, multi-center, and ideally multi-national validation using distinctly mediastinal-targeted endpoints to stage mediastinal lymph nodes using AI-based 18F-FDG PET/CT radiomics. Standardization of the radiomics workflow, including acquisition procedure, segmentation technique, feature harmonization, and reporting standards, is essential to promote reproducibility and enable cross-institutional comparability.

A particularly promising methodological direction is the shift from standard hand-crafted radiomics towards deep learning radiomics (DLR), where convolutional neural networks build discriminative representations from raw PET and CT pixel data without manual feature engineering. DLR has repeatedly demonstrated superior performance in oncologic imaging, detecting sub-visual patterns that are invisible to skilled readers and undetectable by standard radiomics pipelines.

The integration of explainable modeling methodologies can also enhance clinical interpretability and support responsibly directed implementation. Future research should also consider the added clinical utility of AI-assisted nodal evaluation beyond typical diagnostic accuracy metrics, including its impact on invasive staging plans, treatment decision-making, and patient outcomes. Most importantly, future studies should move beyond surrogate endpoints such as AUC and demonstrate real benefits in patient-centered outcomes such as reduction of unnecessary invasive procedures (mediastinoscopy, EBUS-TBNA) and, ultimately, overall and disease-specific survival.

Further retrospective analyses should be avoided in favor of prospective, real-time clinical validation studies with pre-specified statistical analysis plans and prospectively registered protocols. Emerging collaborative frameworks for privacy-preserving distributed model training may further facilitate multinational AI development across geographically and ethnically diverse cohorts while maintaining patient privacy and ensuring compliance with GDPR regulations, thereby addressing the single-country limitation that characterizes the current evidence base.

## 5. Conclusions

Artificial intelligence-based radiomics applied to 18F-FDG PET/CT shows significant promise in improving the mediastinal lymph node staging of non-small cell lung cancer beyond conventional visual and threshold-based evaluation. Based on the available evidence, diagnostic performance is consistently moderate to high, indicating that quantitative imaging analysis captures information not fully represented by conventional PET metrics.

Significantly, model consistency across independent validation cohorts appears to be clinically more important than peak accuracy in development cohorts. Although performance attenuation is observed in external and prospective settings, clinically relevant discrimination is maintained, supporting the feasibility of reproducible clinical implementation.

Multimodal integration of radiomics with clinical and metabolic parameters further enhances prediction, particularly for discriminating false-positive cases of 18F-FDG uptake in the mediastinal compartment.

However, the available evidence remains constrained by methodological heterogeneity and lack of prospective multicenter validation. Standard processes, clear modeling plans, and clearly defined nodal staging that is specifically mediastinal are imperative in determining whether AI-assisted nodal staging truly has clinical value or not.

Bridging the gap between diagnostic accuracy gains and genuine clinical impact will require the adoption of deep learning radiomics, federated multicenter training architectures, and prospective international trials designed to demonstrate measurable improvements in patient survival and reduction of unnecessary invasive staging procedures.

## Figures and Tables

**Table 1:** Quality and risk of bias assessment of the analyzed studies using QUADAS-2.

Study Title	PS	IT	RS	FT
Lai et al., 2025 [14]	Low	Low	Low	Low
Duan et al., 2025 [7]	Low	Unclear	Low	Low
Xie et al., 2021 [29]	Low	Low	Low	Low
Wang et al., 2026 [9]	Low	Unclear	Low	Low
Wdowiak et al., 2025 [30]	Low	Low	Low	Low
Zhong et al., 2023 [26]	Low	Low	Low	Low
Ren et al., 2023 [31]	Low	Unclear	Low	Low
Zhai et al., 2025 [32]	Low	Unclear	Low	Low
Huang et al., 2023 [33]	Low	Low	Low	Unclear
Duan et al., 2025 [15]	Low	Unclear	Low	Low
Dong et al., 2024 [34]	Low	Low	Low	Low
Laros et al., 2022 [35]	Unclear	Low	Low	Unclear
Bi et al., 2026 [36]	Low	Low	Low	Low

PS = patient selection; IT = index test; RS = reference standard; FT = flow and timing. Each domain was evaluated as having low, high, or unclear risk of bias according to the QUADAS-2 recommendations.

**Table 2:** Summary of studies applying AI and radiomics on 18F-FDG PET/CT for lymph node metastasis assessment.

Author, Year	Study Design	Sample Size (n)	Imaging Modality	AI/Radiomics Approach	Target Outcome	AUC	Reference Standard
Lai et al., 2025 [14]	Retrospective cohort	252	18F-FDG PET/CT	PET/CT-based radiomics with supervised ML	Mediastinal LN metastasis	0.95 (validation)	Histopathology
Xie et al., 2021 [29]	Retrospective cohort	263	18F-FDG PET/CT	Radiomics + clinical nomogram	Preoperative LN metastasis	0.828	Histopathology
Wang et al., 2026 [9]	Retrospective cohort	192	18F-FDG PET/CT	TLPC radiomics + ML	LN metastasis; N2 stage	0.93; 0.94	Histopathology
Wdowiak et al., 2025 [30]	Retrospective cohort	87 + 124	18F-FDG PET/CT	ML classifier combining PET/CT + clinical variables	N0/1 vs. N2/3 staging	Not reported	Histopathology
Zhong et al., 2023 [26]	Retrospective + external & prospective validation	1911 + 355 + 999	18F-FDG PET/CT	Cross-modal deep learning (DLNMS)	Occult LN metastasis (N1/N2)	0.958; 0.942; 0.879; 0.875; 0.914; 0.919	Histopathology
Ren et al., 2023 [31]	Retrospective cohort	260	18F-FDG PET/CT	Clinico-biological-radiomics ML	False-positive LN discrimination/LN metastasis	0.90; 0.89	Histopathology
Zhai et al., 2025 [32]	Multicenter retrospective	88	18F-FDG PET/CT	Multimodality fusion radiomics + ML	Mediastinal–hilar LN metastasis	0.950–0.952; 0.923–0.927	Histopathology
Huang et al., 2023 [33]	Retrospective cohort	155	18F-FDG PET/CT	Radiomics signature + nomogram (LASSO LR)	Mediastinal LN metastasis	0.851/0.826; 0.869/0.847	Histopathology
Duan et al., 2025 [7]	Retrospective diagnostic study with external validation	185	18F-FDG PET/CT	PET/CT radiomics + supervised ML (DL-derived features)	Mediastinal–hilar LN metastasis	0.865/0.865	Histopathology
Duan et al., 2025 [15]	Retrospective diagnostic cohort	248	18F-FDG PET/CT	Clinical + radiomics + DL (ResNet50); XGBoost; SHAP	LN metastasis prediction	0.853	Histopathology
Dong et al., 2024 [34]	Multicenter retrospective (3 centers)	381	18F-FDG PET/CT	GBDT-LR (gradient-boosted decision tree–logistic regression)	Confusing mediastinal LN metastasis	0.90	Histopathology
Laros et al., 2022 [35]	Multicenter retrospective (2 centers, Western Europe)	148	18F-FDG PET/CT	XGBoost classifier combining radiomics + clinical variables	Intrathoracic LN classification (malignant vs. benign)	0.97	Histopathology
Bi et al., 2026 [36]	Retrospective cohort	390 (1026 LN stations)	18F-FDG PET/CT	8 ML models compared (LR, CART, SVM, GBDT, RF, MLP, XGBoost, KNN)	Mediastinal LN metastasis	0.93 (XGBoost)	Histopathology

## Data Availability

No new data were created or analyzed in this study. Data sharing is not applicable to this article.

## Supplementary Materials

The following supporting information can be downloaded at: https://www.mdpi.com/article/10.3390/diagnostics16132014/s1, PRISMA 2020 Checklist. Reference [28] is cited in the supplementary materials.

## Abbreviations

The following abbreviations are used in this manuscript:

AI	Artificial Intelligence
AUC	Area Under the Curve
CART	Classification and Regression Tree
CBR	Clinico-Biological-Radiomics
CT	Computed Tomography
DL	Deep Learning
DLR	Deep Learning Radiomics
DLNMS	Deep Learning Nodal Metastasis Signature
EBUS	Endobronchial Ultrasound
ESMO	European Society for Medical Oncology
ESTS	European Society of Thoracic Surgeons
FDG	Fluorodeoxyglucose
18F-FDG	18F-fluorodeoxyglucose
GBDT	Gradient-Boosted Decision Tree
GDPR	General Data Protection Regulation
GLOBOCAN	Global Cancer Observatory
IRB	Institutional Review Board
KNN	K-Nearest Neighbors
LASSO	Least Absolute Shrinkage and Selection Operator
LN	Lymph Node
LNM	Lymph Node Metastasis
LR	Logistic Regression
ML	Machine Learning
MLP	Multilayer Perceptron
MRI	Magnetic Resonance Imaging
NSCLC	Non-Small Cell Lung Cancer
PET	Positron Emission Tomography
PET/CT	Positron Emission Tomography/Computed Tomography
PICO	Population, Index Test, Comparator, Outcome
PRISMA	Preferred Reporting Items for Systematic Reviews and Meta-Analyses
QUADAS-2	Quality Assessment of Diagnostic Accuracy Studies-2
RF	Random Forest
SHAP	SHapley Additive exPlanations
SUV	Standardized Uptake Value
SUVmax	Maximum Standardized Uptake Value
SVM	Support Vector Machine
TBNA	Transbronchial Needle Aspiration
TCIA	The Cancer Imaging Archive
TLPC	Tumor and Lymph Node PET/CT

## References

[1] Sung H., Ferlay J., Siegel R.L., Laversanne M., Soerjomataram I., Jemal A., Bray F. (2021). Global Cancer Statistics 2020: GLOBOCAN Estimates of Incidence and Mortality Worldwide for 36 Cancers in 185 Countries. CA Cancer J. Clin..

[2] Zhou J., Xu Y., Liu J., Feng L., Yu J., Chen D. (2024). Global burden of lung cancer in 2022 and projections to 2050: Incidence and mortality estimates from GLOBOCAN. Cancer Epidemiol..

[3] Díaz J.L., Edwards J., Deleu A.-L., Giaj-Levra N., Prisciandaro E., Roch B., Paesmans M., Berghmans T., Brandão M. (2024). What Does N2 Lymph Node Involvement Mean for Patients with Non-Small Cell Lung Cancer (NSCLC)?—A Review of Implications for Diagnosis and Treatment. Cancers.

[4] Mingels C., Madani M.H., Sen F., Nalbant H., Riess J.W., Abdelhafez Y.G., Ghasemiesfe A., Rominger A., Guindani M., Badawi R.D. (2025). Diagnostic accuracy in NSCLC lymph node staging with Total-Body and conventional PET/CT. Eur. J. Nucl. Med. Mol. Imaging.

[5] Rogasch J.M., Frost N., Bluemel S., Michaels L., Penzkofer T., von Laffert M., Temmesfeld-Wollbrück B., Neudecker J., Rückert J.-C., Ochsenreither S. (2021). FDG-PET/CT for pretherapeutic lymph node staging in non-small cell lung cancer: A tailored approach to the ESTS/ESMO guideline workflow. Lung Cancer.

[6] Valizadeh P., Jannatdoust P., Pahlevan-Fallahy M.-T., Hassankhani A., Amoukhteh M., Bagherieh S., Ghadimi D.J., Gholamrezanezhad A. (2025). Diagnostic accuracy of radiomics and artificial intelligence models in diagnosing lymph node metastasis in head and neck cancers: A systematic review and meta-analysis. Neuroradiology.

[7] Duan F., Zu H., Zhang R., Li Y., Zhao Y., Wang X., Zhang M., Li P., Wang D. (2025). Deep learning radiomics and 18F-FDG PET/CT imaging: Mediastinal lymph node characteristics as predictors of metastasis in non-small cell lung cancer. Transl. Lung Cancer Res..

[8] Lambin P., Leijenaar R.T.H., Deist T.M., Peerlings J., de Jong E.E.C., van Timmeren J., Sanduleanu S., Larue R.T.H.M., Even A.J.G., Jochems A. (2017). Radiomics: The bridge between medical imaging and personalized medicine. Nat. Rev. Clin. Oncol..

[9] Wang H., Hu Q., Tong Y., Zhu H., He L., Cai J. (2026). Chest Computed Tomography-Based Radiomics and Machine Learning for Classifying Mediastinal Lymphadenopathy Caused By Hematologic Malignancies and Metastatic Abdominopelvic Solid Cancers. J. Thorac. Imaging.

[10] Stawarz K., Gorzelnik A., Klos W., Korzon J., Kissin F., Bieńkowska-Pluta K., Stawarz G., Rusetska N., Zwolinski J. (2025). Systematic review of artificial intelligence and radiomics for preoperative prediction of extranodal extension and lymph node metastasis in oropharyngeal cancer. Front. Oncol..

[11] Kandathil A., Kay F.U., Butt Y.M., Wachsmann J.W., Subramaniam R.M. (2018). Role of FDG PET/CT in the eighth edition of TNM staging of non–small cell lung cancer. RadioGraphics.

[12] Yu D., Chen C. (2024). [18F]FDG PET/CT versus [18F]FDG PET/MRI in staging of non-small cell lung cancer: A head-to-head comparative meta-analysis. Front. Med..

[13] Sathekge C., Maes J., Maes A., Van de Wiele C. (2025). FDG PET/CT for Staging Lung Carcinoma: An Update. Semin. Nucl. Med..

[14] Lai R., Geng Y., Sheng D., Ding C., Qian C., Jiang C., Zhou Z. (2025). ^18^F-FDG PET/CT radiomics model from non-small cell lung cancer for preoperative prediction of lymph node metastasis based on overall data and the subset of occult lymph nodes. Hell. J. Nucl. Med..

[15] Duan F., Zhang M., Yang C., Wang X., Wang D. (2025). Non-invasive Prediction of Lymph Node Metastasis in NSCLC Using Clinical, Radiomics, and Deep Learning Features From 18F-FDG PET/CT Based on Interpretable Machine Learning. Acad. Radiol..

[16] Darling G.E., Maziak D.E., Inculet R.I., Gulenchyn K.Y., Driedger A.A., Ung Y.C., Gu C.-S., Kuruvilla M.S., Cline K.J., Julian J.A. (2011). Positron emission tomography-computed tomography compared with invasive mediastinal staging in non-small cell lung cancer: Results of mediastinal staging in the early lung positron emission tomography trial. J. Thorac. Oncol..

[17] Schmidt-Hansen M., Baldwin D.R., Hasler E., Zamora J., Abraira V., I Figuls M.R. (2014). PET-CT for assessing mediastinal lymph node involvement in patients with suspected resectable non-small cell lung cancer. Cochrane Database Syst. Rev..

[18] Konishi J., Yamazaki K., Tsukamoto E., Tamaki N., Onodera Y., Otake T., Morikawa T., Kinoshita I., Dosaka-Akita H., Nishimura M. (2003). Mediastinal lymph node staging by FDG-PET in patients with non-small cell lung cancer: Analysis of false-positive FDG-PET findings. Respiration.

[19] Li M., Wu N., Liu Y., Zheng R., Liang Y., Zhang W., Zhao P. (2012). Regional nodal staging with 18F-FDG PET–CT in non-small cell lung cancer: Additional diagnostic value of CT attenuation and dual-time-point imaging. Eur. J. Radiol..

[20] Qiao J., Zhang X., Du M., Wang P., Xin J. (2022). 18F-FDG PET/CT radiomics nomogram for predicting occult lymph node metastasis of non-small cell lung cancer. Front. Oncol..

[21] Shimada Y., Kudo Y., Maehara S., Fukuta K., Masuno R., Park J., Ikeda N. (2023). Artificial intelligence-based radiomics for the prediction of nodal metastasis in early-stage lung cancer. Sci. Rep..

[22] Hu Q., Li K., Yang C., Wang Y., Huang R., Gu M., Xiao Y., Huang Y., Chen L. (2023). The role of artificial intelligence based on PET/CT radiomics in NSCLC: Disease management, opportunities, and challenges. Front. Oncol..

[23] Chen L., Chen B., Zhao Z., Shen L. (2024). Using artificial intelligence based imaging to predict lymph node metastasis in non-small cell lung cancer: A systematic review and meta-analysis. Quant. Imaging Med. Surg..

[24] Avanzo M., Wei L., Stancanello J., Vallières M., Rao A., Morin O., Mattonen S.A., El Naqa I. (2020). Machine and deep learning methods for radiomics. Med. Phys..

[25] van Leeuwen K.G., Schalekamp S., Rutten M.J.C.M., van Ginneken B., de Rooij M. (2021). Artificial intelligence in radiology: 100 commercially available products and their scientific evidence. Eur. Radiol..

[26] Zhong Y., Cai C., Chen T., Gui H., Deng J., Yang M., Yu B., Song Y., Wang T., Sun X. (2023). PET/CT based cross-modal deep learning signature to predict occult nodal metastasis in lung cancer. Nat. Commun..

[27] Huang X., Huang X., Wang K., Bai H., Ye B., Jin G. (2025). Habitat-based radiomics from contrast-enhanced CT and clinical data to predict lymph node metastasis in clinical N0 peripheral lung adenocarcinoma ≤ 3 cm. Sci. Rep..

[28] Page M.J., McKenzie J.E., Bossuyt P.M., Boutron I., Hoffmann T.C., Mulrow C.D., Shamseer L., Tetzlaff J.M., Akl E.A., Brennan S.E. (2021). The PRISMA 2020 statement: An updated guideline for reporting systematic reviews. BMJ.

[29] Xie Y., Zhao H., Guo Y., Meng F., Liu X., Zhang Y., Huai X., Wong Q., Fu Y., Zhang H. (2021). A PET/CT nomogram incorporating SUVmax and CT radiomics for preoperative nodal staging in non-small cell lung cancer. Eur. Radiol..

[30] Wdowiak A., Rogasch J.M.M., Baumgärtner G.L., Frost N., Rückert J.-C., Neudecker J., Ochsenreither S., Gerhold M., Schmidt B., Graff M. (2025). Independent Validation of a Machine Learning Classifier for Predicting Mediastinal Lymph Node Metastases in Non-Small Cell Lung Cancer Using Routinely Obtainable [^18^F]FDG-PET/CT Parameters. Curr. Oncol..

[31] Ren C., Zhang F., Zhang J., Song S., Sun Y., Cheng J. (2023). Clinico-biological-radiomics (CBR) based machine learning for improving the diagnostic accuracy of FDG-PET false-positive lymph nodes in lung cancer. Eur. J. Med. Res..

[32] Zhai W., Zhou T., Zhou Q., Lin X., Jiang X., Zhang Z., Jin Q., Liu S., Fan L. (2025). A machine learning-based 18F-FDG PET/CT multi-modality fusion radiomics model to predict Mediastinal-Hilar lymph node metastasis in NSCLC: A multi-centre study. Clin. Radiol..

[33] Huang Y., Jiang X., Xu H., Zhang D., Liu L.-N., Xia Y.-X., Xu D.-K., Wu H.-J., Cheng G., Shi Y.-H. (2023). Preoperative prediction of mediastinal lymph node metastasis in non-small cell lung cancer based on 18F-FDG PET/CT radiomics. Clin. Radiol..

[34] Dong S., Fu A., Liu J. (2024). Prediction of metastases in confusing mediastinal lymph nodes based on flourine-18 fluorodeoxyglucose (18F-FDG) positron emission tomography/computed tomography (PET/CT) imaging using machine learning. Quant. Imaging Med. Surg..

[35] Laros S.S.A., Dieckens D., Blazis S.P., van der Heide J.A. (2022). Machine learning classification of mediastinal lymph node metastasis in NSCLC: A multicentre study in a Western European patient population. EJNMMI Phys..

[36] Bi T., Qiang M., Duan X., Yin Y., Zhang W., Chen Z., Zhang X., Ma J., Zhang B., Tang M. (2026). Machine learning-driven PET-CT and clinical pathology model for predicting mediastinal lymph node metastasis in non-small cell lung cancer: A retrospective cohort study. PeerJ.

[37] Ferro A., Bottosso M., Dieci M.V., Scagliori E., Miglietta F., Aldegheri V., Bonanno L., Caumo F., Guarneri V., Griguolo G. (2024). Clinical applications of radiomics and deep learning in breast and lung cancer: A narrative literature review on current evidence and future perspectives. Crit. Rev. Oncol..

[38] Na K.J., Choi H., Kim Y.T. (2020). Radiomics signature for prediction of N2 disease: Fascinating but still a long way to go for clinical application. Transl. Lung Cancer Res..

[39] Hu Y., Zhang Y., Lu Y., Xu Y., Xu J., Zhong H., Cheng L., Zhong R. (2024). Heterogeneity in PD-L1 expression between primary and metastatic lymph nodes: A predictor of EGFR-TKI therapy response in non-small cell lung cancer. Respir. Res..

[40] Li W., Ju L., Zhang S., Chen Z., Li Y., Feng Y., Xiang Y., Xiang T., Wu Z., Pang H. (2025). Research on the Precise Differentiation of Pathological Subtypes of Non-Small Cell Lung Cancer Based on ^18^F-FDG PET/CT Radiomics Features. Cancers.

